# Editorial Notes

**Published:** 1882-12

**Authors:** 


### EDITORIAL NOTES.

HERBERT SPENCER did real missionary work in preaching to us restless Americans the gospel of relaxation. It now behooves physicians here to sanction such teaching, and say amen to the words of this eminent philosopher. If holidays were more frequent, we should hear less of many forms of nervous diseases already too common.

THE Anglo-Swiss Condensed Milk Co. supply the profession with the best and most reliable quality of condensed milk. The value of a certain standard and quality of milk, for the use of infants especially, is a recognized want, which is happily supplied by the article furnished by this company. We recommend it, and base our opinion upon rather an extensive use of the article.

IN a former article we have already made a plea for the introduction and employment of the metrical system by physicians.

The writer has employed it exclusively for a period of nearly two years and is more than satisfied with the change. We regret to see that the adoption has not become more universal. It is a duty which physicians owe to the progress of science, and we hope that this step in advance will no longer be delayed.

We chronicle, with pleasure, the return to our city of Dr. Frederick Peterson from a sojourn of eighteen months in Europe. The interesting correspondence published in the Journal from his pen demonstrates the aptitude of our friend in his professional studies and as a close observer of medical customs and thought in the great medical centres which he visited. We anticipate for Dr. Peterson a brilliant career and an abundant success in his profession.

Differing Doctors at Albany.—As the time draws near for the meeting in Albany, having in view the discussion of the important question in medical ethics, it is evident that there will be such an assemblage of forces on both sides as will serve to thoroughly test the advisability of the recent innovation in a long-established code. An interesting debate may be expected, and the ardent supporters of each faction must whet their swords for a vigorous combat.

If Buffalo physicians realized how instructive are some of the meetings of local scientific societies, they would attend them more frequently. Quite a number are already active members of the Microscopical Club, and consequently topics are frequently brought up, for discussion there, which are intimately related to medicine. It is by thus enlarging the field of study that a man makes his own daily observations, more exactly for his patient and satisfactorily for himself.

The friends of Dr. S. F. Mixer will heartily congratulate him on the pleasant trip he has in prospect to the Pacific slope. It is to be hoped the genial climate of Southern California will prove all the benefit anticipated, and that he will return home refreshed, mentally and physically, by his tour of several months. At the same time such a prolonged absence will leave a void in the medical circle, not easily filled ; though we welcome most warmly to our midst the young physician who assumes his duties during the interval.

We call attention to the interesting correspondence from our editorial confrere who is now enjoying the advantages of rest and study in Philadelphia. The observations made in regard to the wide range and extent of clinical instruction in that great medical centre are ably presented, and, we doubt not, will be attentively perused by the profession. It is becoming more evident that we need not seek foreign advantages for professional improvement, while so much is to be had at home; and we gladly chronicle the portrayal of these facilities from so able a pen.

The New York Polyclinic, modeled after the celebrated Polyclinic of Vienna, has at length been established. If we may judge from rather an enthusiastic editorial in a late number of the *Medical Gazette*, the members of the profession need not seek the advantages of its celebrated prototype, for much superior instruction and instructors are found at home. We join in congratulations for any movement which provides improved facilities for, and an elevation of the standard of medical education. If ably managed, no doubt need be entertained of its success. The profession are prepared for yet greater advances than the schools are inclined to make. This and every other step in our system of medical education will be watched with jealous care, and, if well founded, will receive a liberal support.

The President's Physicians.—The pitiful wrangle in Washington over the expenses of the late President's illness is certainly to be deplored by a right-minded community at large, yet, unfortunately, finds a parallel in almost every physician's experience. Who, in the profession, does not know—when disease has claimed a patient—of the confidence implied, the unbounded gratitude expressed, for services being rendered? And who does not remember how the tone of all interested altered when recovery, or, less happily, death made those services no longer a matter of vital importance? In the case in question, the eminent position occupied by the majority of the medical men is, or ought to be, a sufficient guaranty that the charges were not in undue excess of the usual demand, and should, therefore, be cheerfully recognized by the proper authorities.

